# Formulation development of a live attenuated human rotavirus (RV3-BB) vaccine candidate for use in low- and middle-income countries

**DOI:** 10.1080/21645515.2021.1885279

**Published:** 2021-04-16

**Authors:** Prashant Kumar, Ravi S. Shukla, Ashaben Patel, Swathi R. Pullagurla, Christopher Bird, Oluwadara Ogun, Ozan S. Kumru, Ahd Hamidi, Femke Hoeksema, Christopher Yallop, Julie E. Bines, Sangeeta B. Joshi, David B. Volkin

**Affiliations:** a Department of Pharmaceutical Chemistry, Vaccine Analytics and Formulation Center, University of Kansas, Lawrence, KS, USA; b Batavia Biosciences B.V., Bioscience Park Leiden, Leiden, The Netherlands; c Murdoch Children’s Research Institute, Department of Paediatrics, University of Melbourne, Parkville, Australia; d Department of Gastroenterology and Clinical Nutrition, Royal Children’s Hospital, Parkville, Australia

**Keywords:** Rotavirus, RV3-BB, live virus vaccine, formulation, stability, oral delivery

## Abstract

Formulation development was performed with the live, attenuated, human neonatal rotavirus vaccine candidate (RV3-BB) with three main objectives to facilitate use in low- and middle- income countries including (1) a liquid, 2–8°C stable vaccine, (2) no necessity for pre-neutralization of gastric acid prior to oral administration of a small-volume dose, and (3) a low-cost vaccine dosage form. Implementation of a high-throughput RT-qPCR viral infectivity assay for RV3-BB, which correlated well with traditional FFA assays in terms of monitoring RV3-BB stability profiles, enabled more rapid and comprehensive formulation development studies. A wide variety of different classes and types of pharmaceutical excipients were screened for their ability to stabilize RV3-BB during exposure to elevated temperatures, freeze-thaw and agitation stresses. Sucrose (50–60% w/v), PEG-3350, and a solution pH of 7.8 were selected as promising stabilizers. Using a combination of an *in vitro* gastric digestion model (to mimic oral delivery conditions) and accelerated storage stability studies, several buffering agents (e.g., succinate, adipate and acetate at ~200 to 400 mM) were shown to protect RV3-BB under acidic conditions, and at the same time, minimize virus destabilization during storage. Several optimized RV3-BB candidate formulations were identified based on negligible viral infectivity losses during storage at 2–8°C and −20°C for up to 12 months, as well as by relative stability comparisons at 15°C and 25°C (up to 12 and 3 months, respectively). These RV3-BB stability results are discussed in the context of stability profiles of other rotavirus serotypes as well as future RV3-BB formulation development activities.

## Introduction

Rotavirus (RV) is the leading cause of acute gastroenteritis in children <5 years of age leading to an estimated 215,000 worldwide deaths annually, mainly in the low- and middle-income countries (LMICs).^1^ The rotavirus-attributed morbidity and mortality could be greatly reduced by global implementation of RV vaccination across all national immunization programs as recommended by the World Health Organization (WHO).^2^ Despite the introduction of RV vaccination in the national immunization programs of 94 countries and subnationally in another 6 countries, an estimated 68.4 million infants remain unvaccinated due to limited manufacturing capacity, high vaccine and implementation costs, lower efficacy in LMICs, and potential safety concerns such as intussusception.^3–7^ Two live attenuated oral RV vaccines have been available for over a decade including RotaTeq® (a pentavalent bovine-human reassortant, G1-4, P8]) and Rotarix® (human monovalent strain, G1P[8]), and have shown effective reduction (>80% and >50%, respectively) in disease incidence in high-income and low-income countries. Two additional live attenuated oral RV vaccines produced by Indian manufacturers (Rotavac® G11P10 and ROTASIIL® G1-4 P[9]) have more recently obtained regulatory approval including WHO pre-qualification approval status.^8–15^

Efforts to address current challenges to global implementation of RV vaccines and to enhance RV vaccine efficacy particularly in LMICs, have included the development of new RV vaccine candidates and the exploration of alternative vaccine administration schedules. The lower efficacy of RV vaccines may be attributed to factors common to all vaccines in children in LMICs (such as reduced immune responses, malnutrition and maternal antibodies) or specific to an orally administered vaccine (such as gut microbial environment, gastric acidity and breast milk antibodies). The potential for RV vaccines to protect against the diversity of global RV strains may vary according to the RV genotype present in the vaccine or the vaccine construct (such as monovalent vs re-assortant vaccine, live attenuated virus vaccine vs. inactivated vaccine). It has been proposed that the reduced vaccine efficacy of the current P[8] containing vaccines in Africa (Rotarix®, RotaTeq®) may relate to population differences in the profile of histo-blood group antigens, specially the ABH and Lewis systems. Enteric pathogens, including rotavirus, use histo-blood group antigens expressed on the mucosal epithelia in the first step of attachment and entry process.^16^

RV3-BB vaccine is a naturally attenuated human neonatal RV vaccine developed at Murdoch Children’s Research Institute (MCRI) from the human neonatal RV strain RV3 (G3P[6]), isolated from the stool of an asymptomatic infant in Melbourne.^17^ Clinical trials of RV3-BB vaccine have shown it to be well tolerated and immunogenic in infants when administered in a neonatal administration schedule (0–5 days of age, 6–8 weeks, 12–14 weeks) or infant administration schedule (6–8 weeks, 12–14 weeks, 16–18 weeks) in Indonesian infants, and it was shown to be highly efficacious with 94% of infants protected from severe RV gastroenteritis at 12 months of age and 75% protected at 18 months following administration in a neonatal schedule starting at birth. Administration of a RV vaccine at birth using the novel characteristics of a human neonatal strain before the development of gastric acidity and a complex gut microbial environment could enhance early protection from severe RV gastroenteritis and support completion of full vaccine schedule while minimizing safety concerns due to intussusception.^4,18,19^

As outlined in more detail in the discussion, clinical trials of the RV3-BB vaccine candidate to date have been conducted using an oral three-dose regimen of a frozen liquid formulation.^4^ Prior to oral administration of the thawed liquid vaccine, gastric acid is pre-neutralized by administration of the antacid Mylanta (except for the first-dose given to neonates which does not include a preneutralization step).^20^ Although this initial formulation and administration strategy enabled early phase clinical trials with birth dose strategy, key design goals (i.e., target product profile) of a commercial RV3-BB vaccine formulation to protect infants from rotavirus disease in LMICs included developing a refrigerator stable, liquid formulation of RV3-BB for oral delivery. Such a formulation should be well-tolerated in infants and able to stabilize the virus over the estimated one-hour transit time in the infant stomach without need for preneutralization of gastric acid. By comparison, commercially available rotavirus vaccines (e.g., RotaTeq®, Rotarix®, and Rotavac®) are refrigerator stable, liquid formulations, while ROTASIIL® is a thermostable, lyophilized dosage form requiring reconstitution. Although freeze-drying greatly improves the storage stability of rotaviruses, the simplicity and lower cost of liquid formulations led to our focus of developing a stable, liquid formulation for distribution in the already established vaccine cold chain. In this work, we describe key experimental results from our RV3-BB formulation development efforts to achieve such a target product profile with an emphasis on use in LMICs.

## Materials and methods

### Materials

Biological materials used during this study were secured by Batavia Biosciences, the Netherlands, as part of collaboration agreements with MCRI and PT-BioFarma. The naturally attenuated human rotavirus RV3-BB seed was GMP manufactured (Meridian Life Sciences, Memphis, USA) by MCRI. Bulk Drug Substance (BDS) batches were obtained from either PT-BioFarma, Indonesia or Batavia Biosciences. The RV3-BB assay reference virus standard (produced at Batavia using the RV3-BB virus stock from BioFarma) and MA104 cells were obtained from Batavia Biosciences.

Sucrose, disodium phosphate and sodium dihydrogen phosphate were purchased from EMD-Millipore, USA. Sodium succinate, sodium citrate, magnesium chloride, malic acid and histidine were purchased from Sigma-Aldrich, USA. PEG-3350 was obtained from Spectrum Chemicals, USA. Sodium acetate was procured from Fluka, USA, while disodium adipate was obtained from TCI America. Type I glass vials (2 mL), Flurotec-coated rubber stoppers and aluminum seals were purchased from West Pharm. Inc., USA. TaqMan® Fast Virus 1-Step Master Mix was purchased from Applied Biosystems (ThermoFisher, USA). Rabbit polyclonal anti-sera (R1303) vs. RV strain SA11 (i.e., rabbit anti-SA11 polyclonal antibody) was obtained from the Enteric Diseases Laboratory, MCRI, Australia while Alexa-488 conjugated secondary antibody (Goat anti-Rabbit IgG) was purchased from Thermo-fisher scientific, USA.

## Methods

### Virus quantification

RV3-BB *in vitro* potency quantification was performed using either a Fluorescent Focus Assay (FFA) or a quantitative Reverse-Transcription Polymerase Chain Reaction (RT-qPCR) assay. These assays were performed by infecting confluent MA104 cells monolayers in 96-well plates with required dilution of the test samples (serial dilutions in case of FFA and 50-fold dilution in case of RT-qPCR assay) followed by 18 ± 0.5 h incubation at 37°C. Both FFA and RT-qPCR assays were performed with a RV3-BB assay reference standard (and other assay controls) and the results were expressed as focus forming units per mL (FFU/mL).

For the FFA assay, a previously reported method for RV3-BB was adapted using MA104 cells,^21^ in which the infected cells were fixed using acetone and treated with rabbit anti-SA11 polyclonal (primary antibody against RV viral proteins) and 50 μL Alexa Fluor 488 conjugated goat anti-rabbit IgG (secondary antibody). Manual counting of fluorescent foci was carried out by using Nikon Eclipse Ti-E inverted fluorescence microscope.

For the higher throughput *in vitro* potency RT-qPCR assay, a previously reported method for a pentavalent mixture of bovine-human RV reassortants using a different cell-line was adapted for the RV3-BB virus and MA104 cells.^22^ The infected cells were lysed by freeze-thaw in presence of 0.45% triton X-100. The lysate was diluted 1:10 in ultrapure nuclease-free water and one step RT-qPCR was performed using 5 µL TaqMan® Fast virus 1-Step Master Mix, 0.5µM of each forward (5ʹ-CTG GAT CAA TGG ACA CAC CAT A-3ʹ) and reverse (5ʹ-GCT GCT TCG GTT GGG TAA TA-3ʹ) primer and 0.25 µM double-quenched probe (5ʹ-56-FAM/ACG AAC TCA/ZEN/ACG CGA GAG GAA GT/3IABkFQ/-3ʹ) for amplification of the VP7 cDNA sequence for quantification of the mRNA produced during replication using QuantStudio™ 7 Flex Real-Time PCR System (Applied Biosystems, USA). The RT-qPCR cycling conditions reported previously by Ranheim et al. were used:^22^ Step 1: Hold 45°C for 30 min; Step 2: Hold 95°C for 10 min; and Step 3: 40 cycles 87°C for 20 s, 55°C for 1 min and 15 s. Quantification of viral titer values of RV3-BB test samples in the RT-qPCR assay was performed by using a standard curve of known RV3-BB virus concentration (determined using FFA assay).

### Excipient screening and stability studies with RV3-BB

Concentrated excipient stock solutions were prepared in respective base buffers, pH adjusted and sterile filtered using a 0.22 µm filter (Millipore, USA). Calculated amounts of the excipient stocks were mixed with RV3-BB BDS in 50 mL sterile conical tubes for the preparation of virus formulations at the target log titer (FFU/mL) and targeted excipient concentration (see Supplemental Table S1) in a sodium phosphate, pH 7.0 buffer, unless otherwise specified in the text. Five hundred microliters of each RV3-BB formulation were dispensed in 2 mL glass vials, stoppered with sterilized Flurotec-coated rubber stoppers and crimped with aluminum seals. RV3-BB formulations were prepared aseptically in a class II biosafety cabinet (Labconco, USA).

Initial excipient screening studies with RV3-BB were focused on selection of “hits” from a list of ~50 excipients from a group of sugars, polyols, proteins, polymers, amino acids, osmolytes, metal ions, chelators, cyclodextrins, salts and buffers (See Supplemental Table S1). RV3-BB samples prepared in a base buffer were stressed in the presence and absence of the additives using freeze-thaw (1 freeze-thaw cycle by freezing at −80°C, and thawing at room temperature), storage at room temperature (25°C for 3 days) and agitation (shaking horizontally at 300 rpm at 25°C for 24 h). Viral titer losses for freeze-thaw samples were calculated with respect to RV3-BB in a base buffer, while for thermal and agitation stressed samples, losses were calculated with respect to control RV3-BB samples prepared in the same excipient and incubated for same amount of time at −80°C and 25°C (no shaking), respectively. All samples subjected to freeze-thaw, thermal and agitation stresses, except for low-pH study (see below), were stored at −80°C until analysis, hence were also subjected to 1 freeze-thaw cycle prior to running the RT-qPCR assay.

Excipient “hits” identified from the initial screening studies as RV3-BB stabilizers were further optimized in terms of concentration and effect of combinations by using elevated temperature (25°C for 1 week) and agitation (shaking horizontally at 300 rpm at 25°C for 1, 3 and 5 days) stresses. For the thermal stress study, RV3-BB samples containing indicated levels of additives were incubated at 25°C for 1 week and titer loss calculated by comparing to the same formulation stored at −80°C. RV3-BB samples for agitation studies were prepared in a formulation containing 400 mM succinate, 50% (w/v) sucrose and PEG-3350 (0–1% w/v) in a phosphate buffer at pH 7.8. Titer losses were calculated with respect to control RV3-BB samples (no agitation) prepared in the same excipient mixture and incubated for the same amount of time at 25°C.

For evaluating RV3-BB viral infectivity titer losses in various formulations due to exposure to acidic pH, an *in vitro* gastric acid digestion model was used as described in detail elsewhere^20^ and as adapted from previously reported literature.^11,23^ Briefly, the RV3-BB formulations were tested for their ability to protect against acidic pH stress by adding 4 mL of 0.1 N HCl to 2 mL of RV3-BB containing formulations for 1 h at 37°C in 15 mL sterile conical tubes, and compared with respect to control formulations (without HCl addition). Both RV3-BB *in vitro* potency and solution pH were measured before and after HCl addition for all the formulations as described elsewhere.^20^ The various formulations contained the indicated levels of buffering salts (varying from 0 to 500 mM) in a sodium phosphate buffer, pH 7.5 containing 50% sucrose. RV3-BB formulations containing the same buffering salts (0–500 mM) in sodium phosphate buffer, pH 7.0 containing 30% sucrose were also incubated to 25°C for 1 week and titer losses calculated with respect to control formulations prepared in the same excipient and incubated at −80°C for same amount for time.

For real-time and accelerated stability studies, RV3-BB candidate formulations were examined under frozen (−20⁰C), real time (2–8⁰C) and accelerated conditions (15, 25 and 37⁰C) and titer losses were determined as compared to a −80⁰C control of the same formulation assayed simultaneously. Linear regression of log loss data vs. time was carried out using Origin 2018 (OriginLab Corporation, Northampton, MA, USA) to calculate log loss using average slope (useful for rank ordering of formulations) and at lower 95%CI (to account for both virus instability and assay variability). All RV3-BB samples were analyzed using RT-qPCR assay in quadruplicates, unless specified, and the average log (titer) calculated. Log potency losses for each sample were calculated compared to a frozen, control sample as described in each figure. The total error value for log loss of viral titer was calculated by the following equation, SE(C) = √(SE(A)^2+ SE(B)^2), to account for error propagation. (A) and (B) are standard error values for the log loss measurements of the control and test formulations, respectively, and (C) is the total standard error for the log loss of viral titer.

## Results

### In vitro potency assay development to enable formulation development for RV3-BB vaccine candidate

**Figure 1.**
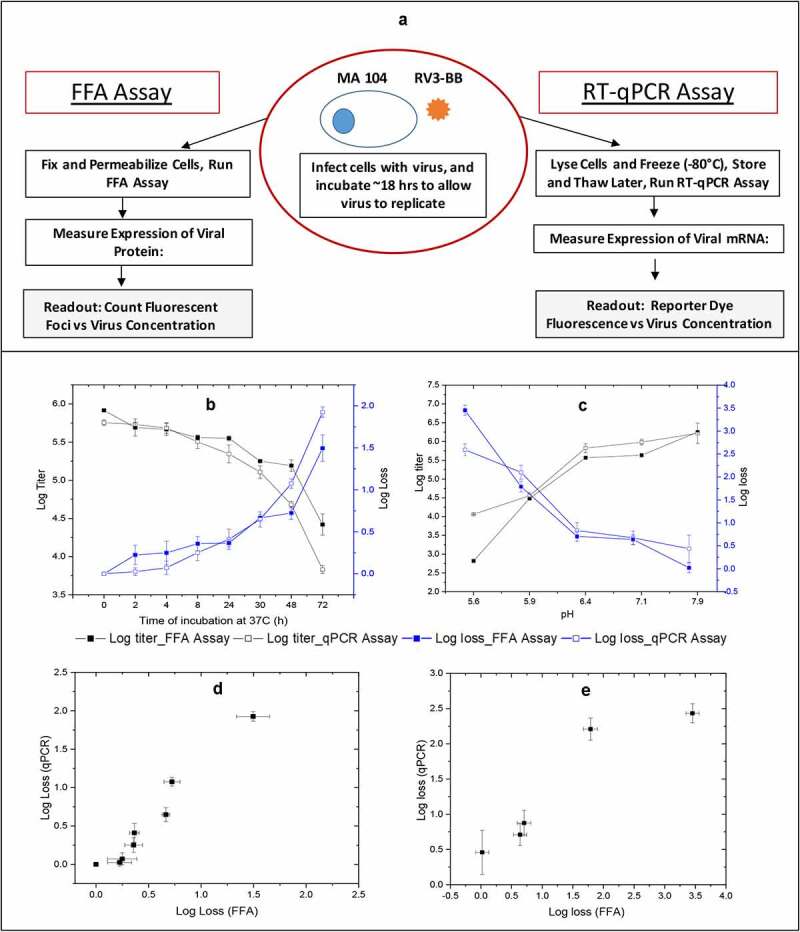
Outline of the FFA and RT-qPCR *in vitro* potency assays and correlations of RV3-BB stability results as measured by both assays. Panel (a) shows outline of the *in vitro* potency assays for determining RV3-BB log titers. The infectious titer (Log FFU/ml) and titer loss (log loss vs. unstressed control) values were measured by RT-qPCR assay and FFA after RV3-BB virus (in DMEM medium) was subjected to (b) Thermal stress at 37°C for up to 72 h, and (c) pH stress at 5.5 to 8.0 at 37°C for 2 h with 20 mM citrate phosphate buffer. Panels (d) and (e) display correlation plots of RV3-BB stability as measured by both *in vitro* potency assays for the thermally-stressed and pH-stressed virus samples, respectively. Data are presented as the mean ± SD (n = 3)

To determine the *in vitro* potency, the Fluorescence Focus Assay (FFA) is the “gold standard” for quantitation of rotaviruses (and other live virus vaccines),^11,14,24^ however, the method is low-throughput, time-consuming, labor-intensive and limited by the availability of primary antibody.^25^ We therefore adapted a high-throughput, 96-well plate infectivity RT-qPCR assay, described previously for live, attenuated bovine-human reassortant RV vaccine with a different cell-line,^22^ for use in RV3-BB formulation development with MA104 cells (see methods). A schematic overview is presented in Figure 1(a); each assay measures RV3-BB expression levels by monitoring either viral protein (FFA) or viral mRNA (RT-qPCR).

To evaluate the ability of the two methods to monitor the stability profile of RV3-BB, virus samples were exposed to elevated temperature (37°C, 72 hr.) or various pH conditions (pH range of 5.5–8.0, 2 hrs.), and infectivity titers were determined by both FFA and RT-qPCR assays. As shown in Figure 1(b,c), RV3-BB *in vitro* potency values (log titer) and stability profile (log loss vs control sample) showed similar results using either assay (Figure 1(b,c)) with RV3-BB progressively losing >90-99% of viral titers (~1.5–2.0 log loss). The correlation of RV3-BB stability results using the FFA versus RT-qPCR assay was evaluated by Pearson’s analysis (r values in the range of 0.1–0.3 indicates small, 0.3–0.5 medium and 0.5–1.0 large positive correlations).^26^ Pearson coefficient r values of 0.98 and 0.98 were calculated for the measured RV3-BB *in vitro* potency losses from the two assays for thermally stressed (Figure 1(d)) and pH stressed (Figure 1(e)) samples, and these results were statistically significant (*p* < 0.05). Based on these comparative RV3-BB stability results, along with no observed assay interference of pharmaceutical excipients (data not shown), the infectivity RT-qPCR assay was implemented in RV3-BB formulation development studies as described below.

### Excipient screening to improve RV3-BB stability during storage

**Figure 2.**
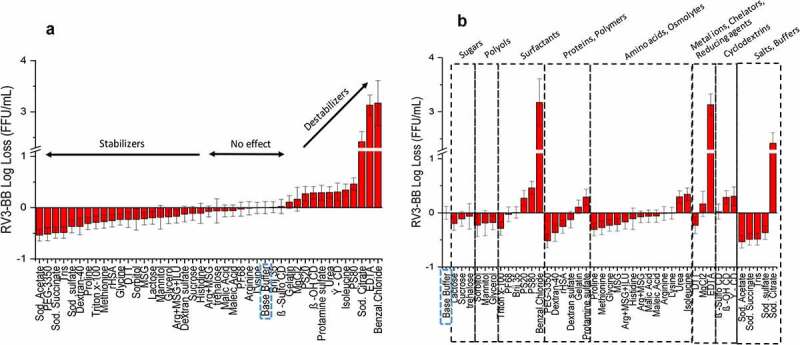
Effect of freeze-thaw on RV3-BB stability in the presence of different excipients as measured by RT-qPCR. (a) Excipients listed in order of protective effect on RV3-BB stability, and (b) Excipients listed by class of additives. Titer losses in individual control formulations (stored at −80⁰C) were calculated with respect to RV3-BB in a no excipient control sample (sodium phosphate buffer at pH 7.0) stored under same conditions to measure the effect of freeze-thaw on RV3-BB stability. The virus log loss titer data are presented as the mean ± SD (n = 3)

A stepwise screening of RV3-BB stability in the presence of ~50 different pharmaceutical excipients was performed to identify potential viral stabilizers against various environmental stresses that may be encountered during manufacturing and long-term storage (i.e., freeze-thaw, elevated temperature, and agitation). Studies were performed in a pH 7.0 buffer (see methods and Supplemental Table S1) in pharmaceutical glass vials due to low virus adsorption and their suitability for use as clinical and commercial dosage forms.^27^ Upon freeze-thaw (F/T) stress, most sugars, polyols, polymers and amino acids stabilized RV3-BB (or had no deleterious effect) as ranked ordered by relative effectiveness (Figure 2(a)) and by excipient category (Figure 2(b)). More complex results were seen with other categories of additives. For example, EDTA, sodium citrate, and benzalkonium chloride resulted in high titer losses (> 2.5 log titer) yet other excipients in the same category stabilized RV3-BB vs. F/T stress.

**Figure 3.**
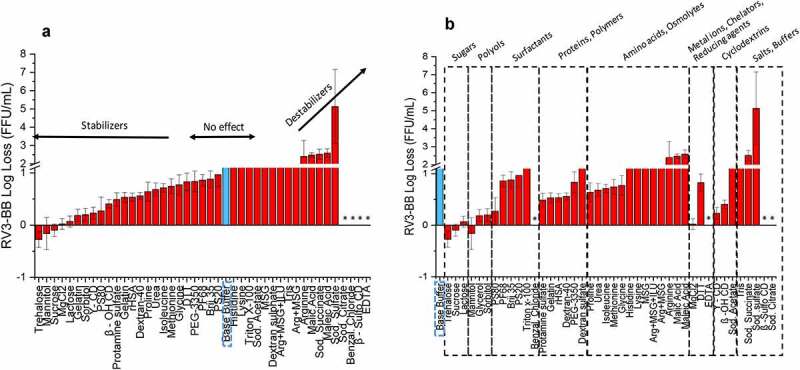
Effect of elevated temperature on RV3-BB stability in the presence of different excipients as measured by RT-qPCR. (a) Excipients listed in order of protective effect on RV3-BB stability, and (b) Excipients listed by class of additives. Loss in viral titers due to thermal stress (3 days in a glass vials at 25°C) was calculated with respect to control samples that were stored at −80°C (prepared with the same excipient in a sodium phosphate buffer, pH 7.0). Excipients showing complete titer loss are indicated by asterisk (*). The virus log loss titer data are presented as the mean ± SD (n = 3)

**Figure 4.**
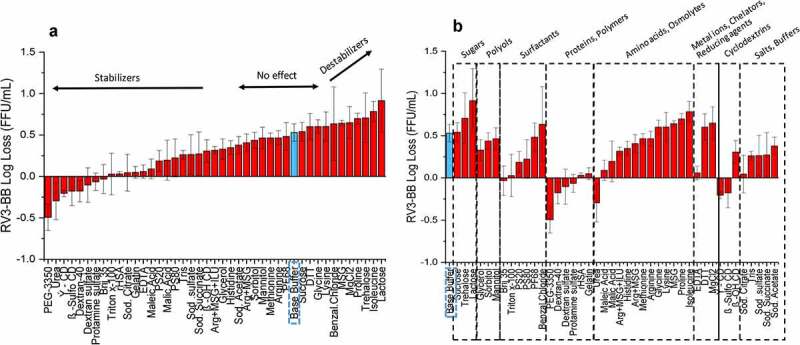
Effect of agitation stress on RV3-BB stability in the presence of different excipients as measured by RT-qPCR. (a) Excipients listed in order of protective effect on RV3-BB stability, and (b) Excipients listed by class of additives. Loss in viral titer due to agitation (300 rpm for 24 h at 25⁰ C) were calculated with respect to the control samples that were stored at 25°C (prepared with the same excipient in a sodium phosphate buffer, pH 7.0), and incubated for the same amount of time without agitation. The virus log loss titer data are presented as the mean ± SD (n = 3)

Similar excipient screening experiments with RV3-BB were performed using other stresses including elevated temperature (Figure 3(a)) and agitation (Figure 4(a)). Thermal stabilization of RV3-BB was observed in the presence of sugars (e.g., sucrose, trehalose, lactose), polyols (e.g., mannitol, glycerol, sorbitol) and proteins (e.g., recombinant human serum albumin, rHSA, and hydrolyzed gelatin) as shown in Figure 3(b). Other categories of additives mostly destabilized the virus including surfactants, amino acids, polymers and salts/buffers, with some interesting exceptions such as MgCl_2_, γ-CD and β-OH-CD, which improved RV3-BB stability. Compounds that resulted in high levels of virus destabilization at elevated temperature included metal chelators (e.g., sodium citrate, EDTA) and charged/sulfated compounds (e.g., sodium sulfate, β-sulfo-cyclodextrin, and benzalkonium chloride). In the case of agitation stress (Figure 4(b)), stabilizing excipient classes for RV3-BB mostly included proteins (e.g., albumin and hydrolyzed gelatin), polymers (e.g., PEG-3350, dextran-40) and surface-active compounds (e.g., beta-sulfo-cyclodextrin, (β-sulfo-CD)).

**Figure 5.**
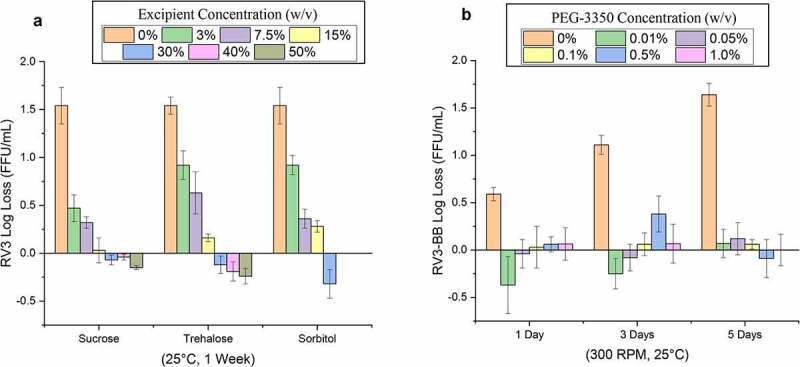
Effect of concentrations of stabilizers on RV3-BB stability during exposure to elevated temperatures and agitation as measured by RT-qPCR. (a) Titration studies of sucrose, trehalose and sorbitol concentration on RV3-BB stability at 25°C for 1 week (at indicated concentration of each additive in a phosphate buffer, pH 7.0). RV3-BB viral titer losses due to thermal stress were calculated with respect to control samples that were stored at −80°C and prepared in the same excipient/base buffer. (b) Titration studies of PEG-3350 concentrations (0 to 1% w/v) on RV3-BB stability upon agitation (300 rpm for 1, 3 and 5 days at 25⁰ C). RV3-BB samples contained 400 mM succinate and 50% (w/v) sucrose in a phosphate buffer at pH 7.8. RV3-BB viral titer losses due to agitation were calculated with respect to their corresponding control samples (incubated for the same amount of time at 25°C without agitation). The virus titer data are presented as the mean ± SD (n = 4)

The most promising RV3-BB stabilizers identified from the screening experiments were then titrated to determine their optimal concentration. For example, at elevated temperatures (25°C for 1 week), sucrose, sorbitol and trehalose stabilized RV3-BB in a concentration-dependent manner (Figure 5(a)) with concentrations ≥ 30% w/v being most effective in stabilizing RV3-BB. During agitation stress (for up to 5 days, 25°C, 300 rpm), titration of PEG-3350 concentration vs RV3-BB stability was performed (Figure 5(b)). The RV3-BB viral titer losses increased as time of agitation increased from 1, 3, and 5 days. When PEG-3350 was added to in a concentration range of 0.01–1.0% (w/v), stabilization of RV3-BB was noted when compared to the control sample (in this case, in a formulation with 50% sucrose (w/v) and 400 mM succinate incubated at 25°C without agitation). The concentration values of the optimal stabilizing effect of PEG-3350 during agitation varied across different RV3-BB bulk preparations (data not shown; see discussion).

A series of similar titration experiments were performed with other “hits” from the excipient screening studies, and the most promising additives (and their concentration ranges) against freeze-thaw, elevated temperature and agitation were identified. In addition, various combinations of promising stabilizers were evaluated as well as the effect of buffer type (histidine and phosphate), metal ions (calcium and magnesium), and solution pH (data not shown). For the final solution pH value, pH 7.8 was shown to be the optimal condition for RV3-BB stability based on accelerated stability studies in the pH range of 6.5–7.8 (data not shown). Based on these optimization experiments, the top conditions for improving RV3-BB viral stability included sucrose (50–60%), PEG-3350 (0.01%), and pH 7.8 in a sodium phosphate buffer as described in more detail below. Although calcium chloride (4 mM) displayed a stabilizing effect, it also resulted in visible precipitation in the formulation and was excluded from further evaluation. The presence of varying volumes of DMEM medium (20–80%) was also evaluated (representing varying titers of viral bulks that may be added to the formulation), and no major effects were observed, although lower DMEM levels trended toward improved viral stability.

### RV3-BB stability in presence of additives that provide buffering capacity at low pH

Two different *in vitro* models were developed to mimic *in vivo* gastric digestion conditions to better understand the stability of RV3-BB virus under varying formulation conditions (types and concentration of additives that have acid-neutralizing capacity, ANC) as well as varying oral delivery conditions (e.g., acid production rates, pre-feeding with infant formula or the antacid Mylanta, final solution pH), as described in detail elsewhere.^20^ In this work, RV3-BB stability was examined in the presence of varying concentrations of down-selected additives (that neutralize gastric acid) under two different, but equally important, conditions for developing a new RV3-BB formulation including (1) the storage stability (25°C for 1 week), and (2) the oral delivery stability using one of the *in vitro* digestion models (4 mL of 0.1 N HCl, 1 hr., 37°C).

**Figure 6.**
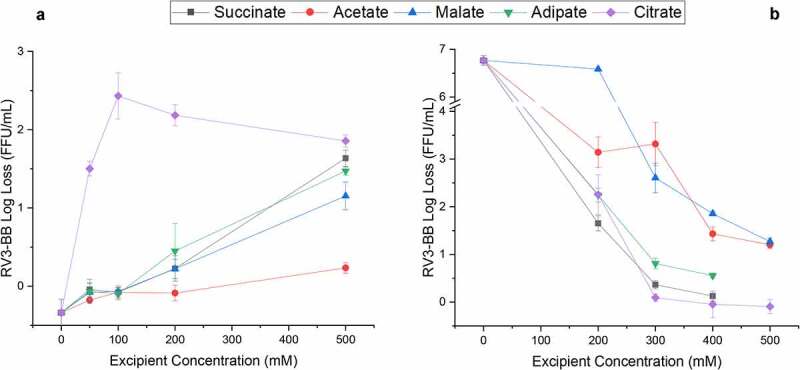
Effect of selected high buffering capacity excipients on RV3-BB stability during exposure to elevated temperatures and acidic pH conditions as measured by RT-qPCR. (a) Log loss RV3-BB viral titers after 1 week at 25°C in formulations containing indicated levels of either succinate, acetate, malate, adipate or citrate (in a phosphate buffer, pH 7.0 containing 30% sucrose). (b) Log loss RV3-BB viral titers after addition of 4 mL 0.1 N HCl (at 37°C for 1 hr.) to RV3-BB formulations containing indicated levels of either succinate, acetate, malate, adipate or citrate (in a phosphate buffer, pH 7.5 containing 50% sucrose). The virus log loss titer data are presented as the mean ± SD (n = 3)

As shown in Figure 6(a), during an accelerated stability study with RV3-BB (at 25°C, 1 week), the buffering salts destabilized RV3-BB in a concentration-dependent manner. For example, the higher the additive concentration (higher buffering capacity), the greater the RV3-BB virus titer loss as measured by infectivity RT-qPCR (Figure 6(a)). However, individual additives had varying effects on RV3-BB stability profiles. Sodium acetate had the least destabilizing effect on RV3-BB, followed by malic acid, adipate and succinate (Figure 6(a)). RV3-BB destabilization was most pronounced in a citrate containing formulation, with over 2 log loss of titer in the presence of 100 mM citrate. In contrast, as shown in Figure 6(b), an opposite trend was observed for RV3-BB stability under acidic pH conditions (as the buffering salts resisted pH change upon HCl addition; data not shown). A complete loss in RV3-BB titer was observed in the absence of these excipients, while succinate (400 mM), citrate (300 to 500 mM) and adipate (400 mM) protected RV3-BB viral titers under these acidic pH conditions (acetate and malate were less protective at all tested concentrations). A more detailed overview of two *in vitro* digestion models (including RV3-BB stability data under various formulation, acidic pH and storage conditions) is presented elsewhere.^20^

In summary, these results demonstrate that excipients with high-buffering capacity stabilize RV3-BB under conditions that mimic oral delivery (by neutralizing acid), but concomitantly, destabilize RV3-BB during accelerated storage conditions. An optimization of these two effects is thus required to design new candidate RV3-BB formulations. Acetate (200–400 mM), succinate (200–400 mM) and adipate (200 mM) were selected as conditions that provided high-buffering capacity to protect RV3-BB during oral delivery yet were relatively the least destabilizing to RV3-BB during accelerated storage conditions.

### Real-time and accelerated stability testing of candidate RV3-BB formulations

Based on the RV3-BB formulation development results described above, various candidate RV3-BB formulations (F1-F9) were selected and prepared as shown in Table 1. A real-time and accelerated stability study of RV3-BB in the nine different formulations (Table 1) was then set up over 12 months at −20, 2–8, 15⁰C, and over 3 months at 25⁰C, and RV3-BB log titers were determined by infectivity RT-qPCR. In addition, −80⁰C frozen samples were prepared and run as controls in the *in vitro* potency assay at each timepoint. The stability of RV3-BB was then reported as a log loss of titer compared to the −80⁰C control, an approach that greatly reduced the effect of assay variability during evaluation of stability profiles of the virus in each candidate formulation. This approach allowed for better comparison of the effect of candidate formulations of RV3-BB and provided an opportunity to better extrapolate stability data using infectivity RT-qPCR to predict longer-term stability trends of RV3-BB in these candidate formulations as described in detail elsewhere.^28^ To compare and rank-order the candidate liquid RV3-BB formulations in this work, we determined the average slope value of viral titer loss over time (at four different temperatures) as measured by infectivity RT-qPCR assays. We also evaluated the lower 95% confidence intervals of the stability data to define potency losses per ICH stability guidelines as described more detail elsewhere.^28^

**Table 1.** Candidate RV3-BB liquid formulations evaluated during accelerated and long-term stability at -20, 2–8, 15, 25°C. A summary of the stability results is also provided in terms or slope of log loss/month of RV3-BB titers at various temperatures. See Figure 7 for corresponding stability data

Form. No.	RV3-BB Average Stability Slope (log loss/month) at -20, 2–8, 15 and 25°C	Additives to stabilize RV3-BB during oral delivery	Additives to stabilize RV3-BB during storage	
			PEG 3350	Sucrose
F1	0.00, 0.01, 0.07, 0.15	0	0.01% (w/v)	60% (w/v)
F2	−0.01, 0.02, 0.05, 0.18	100 mM Succinate		
F3	0.01, 0.01, 0.12, 0.34	200 mM Succinate		
F4	0.00, 0.02, 0.21, 0.99	300 mM Succinate		
F5	0.01, 0.02, 0.25, 0.91	400 mM Succinate		
F6	−0.02, −0.02, 0.05, 0.12	200 mM Acetate		
F7	0.00, 0.02, 0.05, 0.31	400 mM Acetate		
F8	−0.01, 0.02, 0.01, 0.21	200 mM Adipate		
F9	0.00, 0.02, 0.13, 0.42	200 mM Succinate	0	

Candidate RV3-BB formulations F1-F9 contained additives summarized in Table and were prepared in a phosphate buffer at pH 7.8. A volume of 0.5 mL for each formulation was filled in stoppered, 2 mL glass vials. The formulations were analyzed by using RT-qPCR assay and spot checks were carried out by using FFA assay. n = 2 vials per time point and temperature.

**Figure 7.**
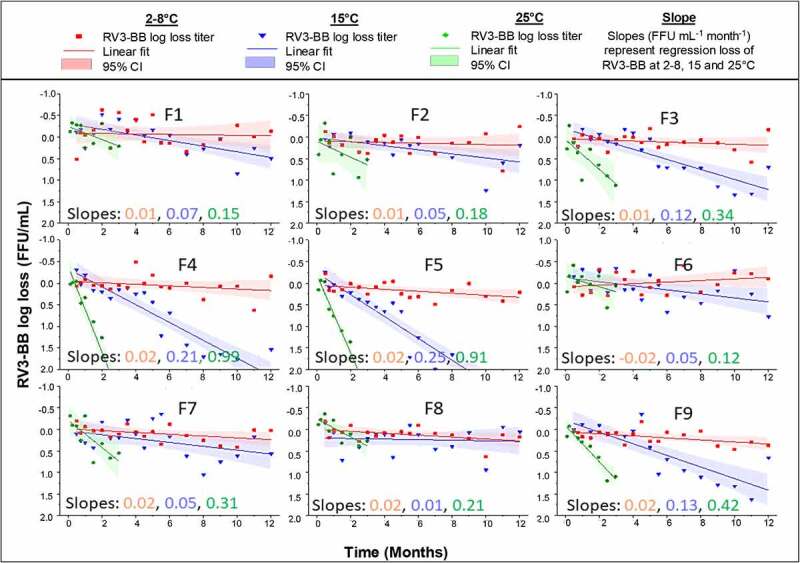
Real-time and accelerate storage stability study of RV3-BB in candidate liquid formulations (F1-F9) over 12 months at 2–8 C, 15 and 25⁰C as measured by RT-qPCR. Composition of each RV3-BB candidate formulation (F1-F9) is shown in Table 1. Solid lines (slopes with units FFU mL-1 month-1) represent regression of mean log loss of RV3-BB viral titer at different temperatures vs. −80⁰C control formulation. Shaded bands represent 95% CI of the regression line accounting for assay variability. The virus log loss titer data are presented as the mean ± SD (n = 4)

As shown in Figure 7, the real-time (2–8⁰C) and accelerated (15, 25⁰C) stability data for RV3-BB in the nine different candidate formulations in terms of log titer loss (compared to a −80⁰C control) is summarized. The average slope values (average slope of log loss titer per month) at the four temperatures (i.e., −20, 2–8, 15, 25⁰C vs. a − 80⁰C control) is also provided in Table 1. All nine candidate RV3-BB formulations displayed excellent stability profiles when stored frozen at −20⁰C or as a liquid formulation at 2–8⁰C. Essentially no losses in viral titers were observed over 1 year at −20⁰C with values of −0.02 to 0.01 loss log/month across the nine candidate formulations (Table 1). Similarly, over 1 year at 2–8⁰C, excellent RV3-BB stability was observed with minimal slope values of −0.02 to 0.02 loss log/month (Table 1 and Figure 7) which converts to an average loss ranging from essentially zero to 0.3 log loss of RV3-BB titer per year. When accounting for assay variability (by considering the lower 95% CI of the slopes) at the one-year timepoint (Figure 7), 0.3 to 0.5 log loss of RV3-BB titer was observed after 1 year of storage at 2–8⁰C. In summary, all of the RV3-BB candidate formulations show similar overall stability profiles with minimal potency losses at −20⁰C and 2–8⁰C (−0.02 to 0.02 loss log/month), with ~0.3 to 0.5 log loss of RV3-BB titer at the lower 95% CI of the stability data after 1 year of storage at 2–8⁰C, which accounts for assay variability from the infectivity RT-qPCR assays as per ICH stability guidelines.^29^ Spot checks using FFA assay at different time points matched with the obtained results using RT-qPCR (data presented elsewhere).^30^

Since all nine candidate RV3-BB formulations displayed excellent overall stability at −20 and 2–8⁰C over 1 year, the ability to differentiate and rank-order the formulations required evaluation of accelerated stability data at 15 and 25⁰C over 1 year and 3 months, respectively (Figure 7). First, no notable effect on RV3-BB storage stability was observed when 0.01% w/v PEG-3350 (F3 vs F9) was excluded from one of the candidate formulations (see Discussion). Second, as the succinate concentration in the candidate formulations increased from zero to 100, 200, 300 and 400 mM (i.e., formulations F1, F2, F3, F4 and F5, respectively), the RV3-BB accelerated storage stability steadily decreased. For example, over 1 year at 15⁰C, viral titer losses increased from 0.07, 0.05, 0.12, 0.21 up to 0.25 log loss/month when comparing formulations F1 to F5, respectively (Table 1 and Figure 7). Similar trends in decreasing RV3-BB stability vs. increasing succinate concentration were observed at 25⁰C over 3 months with values ranging from 0.15 up to 0.99 loss log/month. Finally, when comparing succinate, acetate, and adipate at 200 mM, overall similar trends in RV3-BB stability was observed with succinate perhaps trending toward slightly higher losses (e.g., ~0.3 vs ~0.2 log loss/month at 25⁰C). In summary, at accelerated temperatures, the nine candidate formulations can be ranked ordered, and it was observed that increasing the concentration of additives with high-buffering capacity (i.e., succinate, acetate or adipate which improve RV3-BB stability upon exposure to low pH), destabilized RV3-BB during storage at elevated temperatures (15 and 25⁰C).

## Discussion

The RV3-BB rotavirus vaccine development program is focusing on a low-cost,^30^ safe and efficacious RV vaccine for protection against RV strains more prevalent in the LMICs.^31^ Further, RV3-BB is capable of replicating in neonatal gut asymptomatically, thus making it suitable for neonatal administration.^4,32^ The birth dose strategy using the neonatal RV3-BB strain allows the possibility for early protection (administering RV vaccine before primary RV infection) of infants against RV infection in low-income settings^4,33^ and also minimizing the risk of intussusception and avoiding potential interference from maternal antibodies.^18,19,34^ Thus, a birth dose strategy may improve the effectiveness and safe implementation of RV vaccination in low-income countries.^32^

Ongoing Phase 2 clinical trials examining various doses of the RV3-BB vaccine candidate are evaluating safety and efficacy against a wide range of circulating RV strains associated with human disease in the LMICs (i.e., G1P[8], G2P[4], G3P[8], G4P[8], G9P[8] and G12P[8]).^33,35,36^ RV3-BB clinical trials have been conducted with a three-dose regimen of a thawed, frozen liquid formulation, preceded by administration of Mylanta to neutralize gastric acid (note the first dose given to neonates excluded preneutralization with the antacid). Various doses of RV3-BB titer administered in clinical trials have been quantified by the “gold standard” FFA potency assay (virus infectivity titer in units of FFU/mL). For example, phase I study conducted with a 1 mL dose of 8.3 × 10^6^ FFU/mL was well tolerated in adults, children and infants.^37^ A phase IIA trial conducted at this same titer (8.3 x 10^6^ FFU/mL) showed the vaccine to be immunogenic and well tolerated in neonates and infants using a three-dose regimen.^32^ A recent phase IIB clinical trial conducted in Indonesia (at a dose of 8.3 × 10^6^ to 8.7 × 10^6^ FFU/mL) showed RV3-BB to be efficacious in neonates and infants, with no intussusception.^4^

The RV3-BB clinical formulation is frozen and consists of 1 mL volume of virus in tissue culture medium supplemented with 10% sucrose.^4^ If commercialized for use in LMICs, this frozen liquid formulation would require thawing and a separate preneutralization step with Mylanta prior to oral administration (for two of the three doses; see above), which would lead to higher vaccine costs as well as major practical challenges with vaccine distribution and administration. Hence, developing a new RV3-BB formulation with an improved target product profile is a critical goal for the RV3-BB rotavirus vaccine development program to enable widespread use in LMICs, i.e., low-cost (≤$3.5 per course of 3 doses),^30^ refrigerator stable, orally delivered, small-volume liquid formulation with sufficient buffering capacity to avoid the need for preneutralization of gastric acid.

### In vitro potency assay for formulation development of RV3-BB vaccine candidate

As a first step to facilitate RV3-BB formulation development work, we compared the stability profile of the RV3-BB vaccine using the “gold standard” FFA viral infectivity assay vs. a rapid, higher throughput infectivity RT-qPCR assay. The analytical readout for FFA assay is infectious viral particles, while for the surrogate RT-qPCR assay, it is mRNA amplified from infectious viral particles. The virus quantification using RT-qPCR assay was accomplished using a standard curve of known virus titer (determined using FFA assay). The stability profile of RV3-BB virus using RT-qPCR assay correlated well with the FFA assay as confirmed by accelerated stability data (Figure 1) and by comparison of real-time stability data.^28^ Compared to the FFA assay, the RT-qPCR assay showed ~4 fold higher throughput, offered notable labor and time savings, as well as increased flexibility (to hold plates at −80°C and analyze samples later at a single dilution), and thus was implemented for use in RV3-BB formulation development work described in this study.

### Excipient selection to improve the stability of RV3-BB vaccine candidate during long-term storage

As a second step to achieve the target product profile goals (see above) for a new RV3-BB formulation, systematic excipient screening and concentration optimization studies were performed with the goal of stabilizing the RV3-BB virus against elevated temperatures, freeze-thaw, agitation, and low-pH stresses. Several sugars and polyols at high concentrations greatly stabilized RV3-BB during storage at elevated temperatures including sucrose, trehalose and sorbitol. Sucrose was selected as a key stabilizer for RV3-BB owing to its high stabilizing effect, low cost, and potentially superior palatability (taste) properties for an oral vaccine. As shown in Figure 5, high concentrations of sucrose, as were selected for use in the new candidate formulations (e.g., 50–60% w/v), notably improved RV3-BB stability compared to the sucrose level in the currently used, frozen liquid clinical formulation (10% w/v). Sugars and polyols are known to stabilize proteins in solution, especially at these high excipient concentrations, due to a preferential hydration mechanism that enhances the conformational stability of proteins.^38^ Sucrose has been shown previously to stabilize rotavirus, and it is used in commercial formulations of other live oral RV vaccines including RotaTeq®, Rotarix® and Rotavac® at 50%, 71% and 7.4% w/v, respectively.^9–11,15^ We had concerns that 60% sucrose may cause instability during long-term storage at −20⁰C (when compared to storage at −80⁰C or 2–8⁰C), due to incomplete freezing leading to bulk water ice crystal formation and local changes in excipient concentration, which can potentially lead to structural destabilization of the virus. Thus, the effect of long-term storage at −20⁰C on the stability of RV3-BB in candidate formulations with high sucrose concentrations was examined. Since no viral titer losses after 12 months were observed, these results demonstrate it is possible to store these candidate RV3 formulations at −20°C, as an alternative to storage at 2–8°C, if frozen storage is required.

Several protein excipients as well as nonionic surfactants stabilized RV3-BB against agitation (e.g., vigorous shaking in glass vials) including rHSA, hydrolyzed gelatin, β-sulfo-cyclodextrin, Pluronic F-68, and PEG-3350. These types of surface-active excipients are known to stabilize proteins during agitation by outcompeting for the air–water interface and preventing surface-induced denaturation of proteins.^38^ Since RV3-BB liquid formulations could be shipped to various countries worldwide, there is potential for agitation during transit. In addition, shearing and mechanical stresses could potentially be inadvertently introduced during manufacturing and fill/finish. PEG-3350 was selected as the preferred agitation stabilizer for RV3-BB due to its superior stabilizing effects (during shaking studies; Figure 5(b)) and low-cost. In addition, PEG-3350 did not affect the long-term, 12 month stability profile of the candidate RV3-BB formulations (in the absence of shaking; Figure 7). Although RotaTeq® contains 0.01% (w/v) polysorbate 80, Rotarix® and Rotavac® do not contain nonionic surfactants or protein additives in their formulations.^10,11,15^ The current study showed 0.01% (w/v) PEG-3350 to be optimal for protecting RV3-BB from agitation stress using one bulk preparation of the virus. However, different RV3-BB bulks produced using different processes showed variation in their agitation stability in presence of PEG-3350 with some bulks showing different losses due to agitation (data not shown). In this work, we selected an optimal concentration to stabilize this RV3-BB bulk vs. agitation. Further optimization of PEG-3350 in the range of 0.01 to 1.0% (w/v) with final RV3-BB bulk process for large-scale production will be required in the future.

Divalent metal ions such as Ca^2+^, Mg^2+^ and Zn^2+^ have been previously shown to stabilize RV formulations as they support viral protein function and stability.^39^ These additives were not included in the RV3-BB formulations, however, due to limited solubility within their effective ranges using these candidate formulations (data not shown). It is likely that sufficient levels of divalent cations that are present in the RV3-BB viral bulks in the DMEM tissue culture medium (which occupies 25% of the volume of the vaccine) to sufficiently stabilize the virus, although additional work is required to confirm this hypothesis.

The addition of EDTA and sodium citrate displayed the most destabilizing effect on RV3-BB viral titer losses amongst all studied excipients. These two compounds have been shown to chelate metal ions that are required for function and stability of RV surface proteins (VP4 and VP7), and infectivity of several RV strains.^10,40,41^ Interestingly, citrate is also one of the high-buffering capacity additives that can neutralize gastric acid. In this regard, citrate can concomitantly act as a RV3-BB destabilizer during storage (Figure 6(a)), but as a RV3-BB stabilizer during exposure to low pH (Figure 6(b)) (i.e., *in vitro* conditions designed to mimic *in vivo* oral delivery) as discussed in more detail below.

### Excipient selection to improve the stability of RV3-BB vaccine candidate to acidic pH encountered during oral administration

Balancing the RV3-BB formulation’s stability during storage at pH 7.8 vs exposure to acidic pH stability during oral delivery was the third key step for development of a new RV3-BB liquid formulation without the need for pre-neutralization of gastric acid. The RV3-BB strain is likely more acid labile as compared to other RV vaccine strains since it requires a higher formulation pH of 7.8 for maximum stability as compared to RotaTeq®, Rotarix® and Rotavac® which are formulated at pH 6.2, 6.3 and 7.2, respectively.^10,11,15^ To this end, succinate (in the range of 100–400 mM), acetate (200–400 mM) and adipate (200 mM) were evaluated in real-time and accelerated stability studies as a criterion for selecting top candidate RV3-BB formulations. In comparison, RotaTeq® and Rotarix® contain 0.1 M phosphate, 0.2 M citrate and 0.47 M sodium adipate, respectively, as acid neutralizing excipients as part of their commercial formulations.^10,11,15^ Rotavac®, on the other hand, uses pre-neutralization with citrate bicarbonate buffer prior to vaccine administration.^15^ Hence, in contrast to the rotaviruses used in other commercial vaccines, RV3-BB behaves differently not only in terms of instability in the presence of citrate (see above), but also as a function of formulation’s optimal solution pH to provide best stability during storage.

Our results demonstrate a careful optimization of excipient concentration required to maximize buffering capacity for administration (RV3-BB stability during oral delivery) and minimize RV3-BB titer losses during storage. For example, under these experimental conditions with RV3-BB, lower concentrations of acetate, adipate, succinate and malic acid (i.e., at a concentration of 200 mM) as buffering salts would result in reduced virus titer loss during accelerated stability (titer log loss ≤ 0.5). A modified *in vitro* gastric acid digestion model has been developed to further estimate target concentrations of succinate in the presence and absence of meal/infant formula as described elsewhere.^20^

The RV3-BB candidate formulations F3 and F9 (Table 1) containing succinate showed a negligible loss of RV3-BB potency at −20°C and only small losses at 2–8°C after 12 months of real-time stability data (0.2–0.3 logs based on average slope, and 0.3–0.4 logs based on lower 95% CI of the slopes). Additionally, RV3-BB candidate formulation F7 (Table 1) containing acetate was also a top RV3-BB candidate formulation and displayed negligible titer loss at −20°C and only small losses at 2–8°C after 12 months (0.3 logs from average slope and 0.4 log based on lower 95% CI of the slope). The addition of 400 mM acetate in formulation F7 was equivalent to 200 mM succinate in formulations F3 and F9 in terms of buffering capacity upon exposure to acidic pH (refer to Figure 6). Finally, in the “best case” scenario, if no additional buffering agent is required for oral administration of the RV3-BB, formulation F1 (with no succinate or acetate) shows excellent storage stability after 12 months at 2–8°C with minimal losses (log loss of 0.1 log based on average slope and 0.4 logs based on lower 95% CI of the slope). Thus, in this study, key formulation parameters affecting RV3-BB stability have been identified, and RV3-BB has been shown to be stable at 2–8°C in various liquid formulations suitable for oral delivery of the vaccine candidate.

In this work, formulation conditions were identified and optimized to stabilize the live, RV3-BB rotavirus vaccine candidate against various stress conditions (i.e., freeze-thaw, elevated temperatures, agitation, and exposure to acidic pH) as well as during long-term storage. The allowable virus potency loss during long-term storage, however, remains unknown and awaits the results of ongoing dose-ranging clinical trials to establish the highest dose that is safe and the lowest dose that is efficacious. This “stability window” will help determine the acceptable stability losses during storage when combined with other factors including analytical considerations (i.e., variability of final QC assay to measure viral infectivity) and regulatory requirements for vaccine expiry dating (i.e., using the lower 95% confidence interval of the stability regression line).^29^ Moreover, the overall cost of the vaccine will depend on several inter-related factors including the clinically required vaccine dose, the virus stability profile during storage, and the achievable viral titers in the bulk manufacturing process. Based on these considerations, the selection of the final RV3-BB formulation awaits results from the ongoing clinical trials and bulk process development activities.^30^

In conclusion, the novel human neonatal RV vaccine, RV3-BB, has the potential to address some of the remaining challenges to global implementation of a safe and efficacious RV vaccine and provide protection from severe RV disease from birth. Candidate liquid formulations have been identified that could achieve a RV3-BB vaccine targeted for use in LMICs that is stable at 2–8°C, does not require preneutralization of gastric acid during oral delivery, and can be produced at low cost at large scale. In meeting these formulation requirements, RV3-BB has the potential to make a significant contribution to reducing the global burden of RV gastroenteritis, particularly in LMICs where many deaths due to RV disease still occur.

## Supplementary Material

Supplemental Material
