# Supplementary material for: Formulation development of a live attenuated human rotavirus (RV3-BB) vaccine candidate for use in low- and middle-income countries
Source: Hum Vaccin Immunother. 2021 Apr 16;17(7):2298–310. doi: 10.1080/21645515.2021.1885279 (PMC8189091; doi:10.1080/21645515.2021.1885279)
Supplement: Supplemental Material [file KHVI_A_1885279_SM1539.docx]

**Supplemental Table S1.** Excipient screening for identification of potential stabilizers of RV3-BB.

| **Category** | **Excipients** | **Selection of hits (against freeze-thaw, thermal and agitation stress)** |
| --- | --- | --- |
| **Sugars** | Sucrose | 30% |
|  | Trehalose | 30% |
|  | Lactose | 10% |
| **Polyols** | Sorbitol | 10% |
|  | Mannitol | 10% |
|  | Glycerol | 10% |
| **Surfactants** | PS-80 | 0.01% |
|  | PF-68 | 0.01% |
|  | Polysorbate -20 | 0.01% |
|  | Brij-35 | 0.01% |
|  | Triton X-100 | 0.01% |
|  | Benzalkonium chloride | 0.01% |
| **Proteins and Polymers** | HSA (recombinant) | 1% |
|  | Gelatin (Hydrolyzed) | 1% |
|  | Protamine sulfate | 0.2% |
|  | Dextran sulfate | 5.0% |
|  | Dextran 40 | 5.0% |
|  | PEG-3350 (not in FDA inactive list) | 5.0% |
| **Amino acids and Osmolytes** | Arginine | 0.1M |
|  | Monosodium glutamate | 0.1M |
|  | Glycine | 0.1M |
|  | Histidine | 0.1M |
|  | Isoleucine | 0.1M |
|  | Lysine | 0.1M |
|  | Proline | 0.1M |
|  | Maleic Acid | 0.1M |
|  | Malic Acid | 0.1M |
|  | Methionine | 0.1M |
|  | Urea | 0.1M |
|  | Arginine + MSG (not in FDA inactive list) | 0.05M+0.05M |
|  | Arg + Glu + Ilu (not in FDA inactive list) | 0.045+0.045+0.01M |
| **Metal ions, Chelators and Reducing agents** | DTT | 1mM |
|  | EDTA | 1mM |
|  | Magnesium chloride | 10mM |
| **Cyclodextrins** | Gamma-Cyclodextrin | 10.0% |
|  | 2-OH propyl b-CD | 10.0% |
|  | Sulfobutyl ether beta-cyclodextrin | 10.0% |
| **Salts and Buffers** | Sodium citrate | 100mM |
|  | Sodium acetate | 100mM |
|  | Sodium sulfate | 100mM |
|  | Sodium succinate | 100mM |
|  | Tris (TromeThamine) | 100mM |

The RV3-BB formulations were prepared at target log titer (FFU/mL) and target excipient concentration as shown in the table above. All above excipients are GRAS and FDA inactive ingredient compliant except, Brij-35 (Non-GRAS & not in FDA inactive list), Triton X-100 (Non-GRAS & not in FDA inactive list), Dextran sulfate (Non-GRAS & not in FDA inactive list) and DTT (Non-GRAS).
